# Molecular investigation of coexistent chronic myeloid leukaemia and peripheral T-cell lymphoma – a case report

**DOI:** 10.1038/srep14829

**Published:** 2015-10-06

**Authors:** Alicja M. Gruszka, Cristina Rabascio, Laura Cannella, Simona Sammassimo, Giovanna Andreola, Giuliana Gregato, Mario Faretta, Angelica Calleri, Rita De Molfetta, Giancarlo Pruneri, Francesco Bertolini, Myriam Alcalay

**Affiliations:** 1 Department of Experimental Oncology European Institute of Oncology, Milan, Italy; 2 Laboratory of Hematology-Oncology, European Institute of Oncology, Milan, Italy; 3 Division of Hemato-oncology European Institute of Oncology, Milan, Italy; 4 Division of Pathology, European Institute of Oncology, Milan, Italy; 5 Universita’ degli Studi di Milano, Milan, Italy

## Abstract

Chronic myeloid leukemia (CML) is a myeloproliferative neoplasm underlain by the formation of BCR-ABL1 – an aberrant tyrosine kinase – in the leukaemic blasts. Long-term survival rates in CML prior to the advent of tyrosine kinase inhibitors (TKIs) were dismal, albeit the incidence of secondary malignancies was higher than that of age-matched population. Current figures confirm the safety of TKIs with conflicting data concerning the increased risk of secondary tumours. We postulate that care has to be taken when distinguishing between coexisting, secondary-to-treatment and second in sequence, but independent tumourigenic events, in order to achieve an unbiased picture of the adverse effects of novel treatments. To illustrate this point, we present a case of a patient in which CML and peripheral T-cell lymphoma (PTCL) coexisted, although the clinical presentation of the latter followed the achievement of major molecular response of CML to TKIs.

## Introduction

Chronic myeloid leukaemia (CML) is clonal stem cell disorder driven by the *BCR-ABL1* fusion gene resulting from a reciprocal translocation between chromosomes 9 and 22 (the Philadelphia (Ph) chromosome) that gives rise to an aberrant chimeric tyrosine kinase (TK) (reviewed in^1^). Most patients are diagnosed in the chronic phase (CP) characterised by hepatosplenomegaly, thrombocytosis, and increased white blood count (WBC) made up of mature granulocytes and their precursors (reviewed in^1^). Until relatively recently, the natural disease history evolved from the CP – usually lasting several years – to the ultimately fatal accelerated (AP) and blast (BP) phases. The development of the TK inhibitors (TKIs) has allowed for the achievement of major molecular response (MMR) and long-term disease control^2^. A small percentage (<5%) of cases treated with TKIs for CML develops second cancer^3^. These include mostly skin comprising melanoma, prostate and gut tumours^3^,^4^. Second haematological tumours in these series of patients are rare albeit exist^3^,^4^. On the other hand, cases of concurrent CML plus another haematological disorder (or even two^5^), such as myeloma or B-cell non-Hodgkin lymphoma (NHL), have been reported^6^,^7^ (and references therein). An exact distinction between a secondary and coexisting malignancy may prove problematic. Here we report a case of CML diagnosed in CP, in which the treatment with imatinib led to the disclosure of lymphocytosis, later identified as peripheral T-cell lymphoma (PTCL). Molecular analyses showed the presence of the lymphoma cells in the diagnostic sample taken at the time of CML onset arguing for the coexistence of the two disorders.

### Case Report

**Figure 1:**
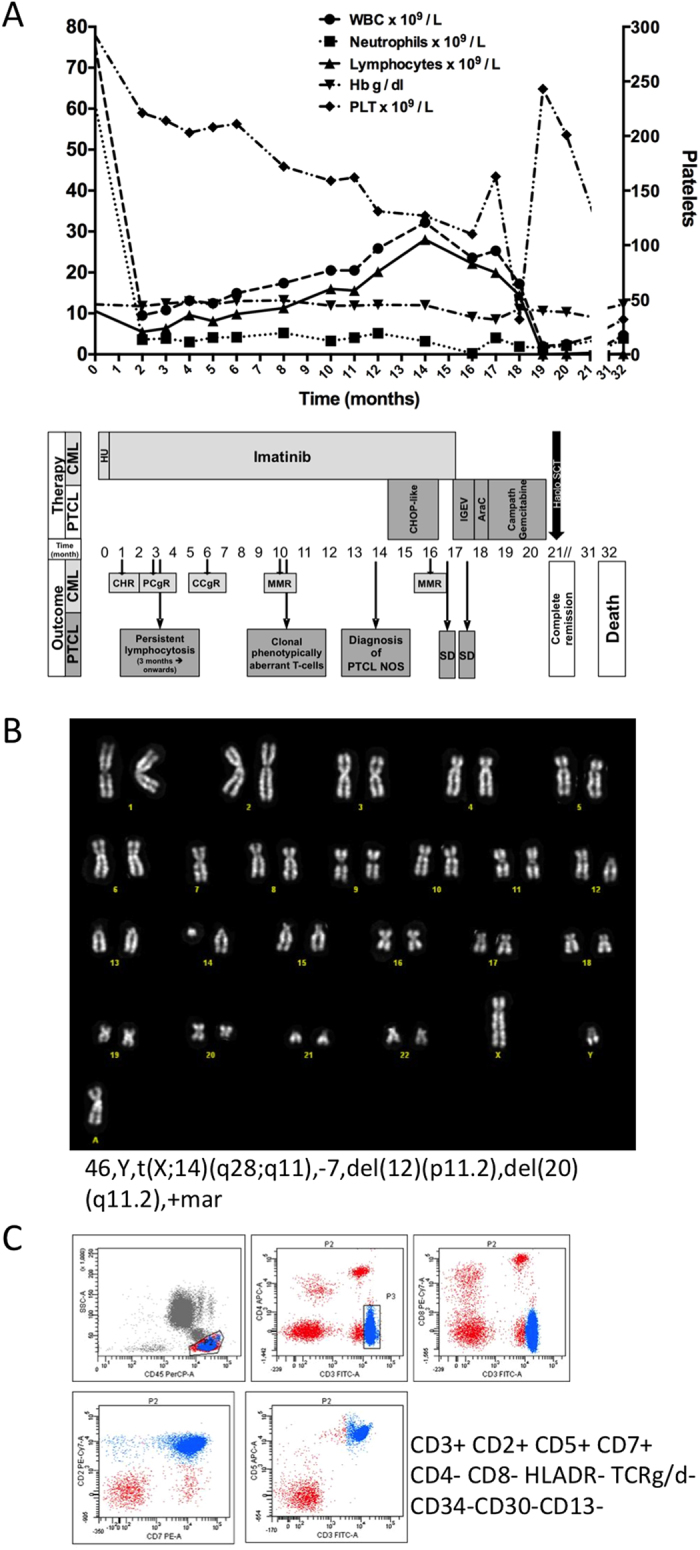
(**A**) Clinical and treatment history of the patient. Hb – haemoglobin, PLT – platelets, WBC – white blood count, HU – hydroxyurea, CHOP - cyclophosmamide, hydroxydaunorubicin, oncovin (vincristine), prednisone, IGEV - ifosfamide, gemcitabine, vinorelbine, CHR (complete haematological response), PCgR – partial cytogenetic response, CCgR – complete cytogenetic response, MMR – major molecular response, SD – stable disease, PTCL NOS – peripheral T-cell lymphoma not otherwise specified. (**B**) A representative karyotype obtained by culturing peripheral blood T-cells and staining the resulting metaphases with the standard Q-banding technique. (**C**) Immunophenotype of the peripheral blood T-cells showing the presence of an aberrant clone.

A 55-year-old man presented with leucocytosis (Hb 12.2 g/dL, Plt 292 × 10^9^/L, WBC 75.5 × 10^9^/L) and splenomegaly. A peripheral blood (PB) and bone marrow (BM) morphological examination were consistent with the chronic phase of a myeloproliferative disorder. Conventional cytogenetic analysis of BM revealed a normal male karyotype with the presence of Ph chromosome in 21/21 metaphases. Quantitative reverse transcription polymerase chain reaction (qRT-PCR) using the *ipsogen* BCR-ABL1 Mbcr IS-MMR Kit (Qiagen, Hilden, Germany) detected existence of the p210 fusion transcript and the *BCR-ABL1/ABL1* ratio of 106.25% was determined. The diagnosis of low-risk (according to Sokal score) Ph+ CML in chronic phase (CML-CP) was thus made. After an initial cytoreduction using hydroxyurea for two weeks, standard dose imatinib treatment was initiated. Within 3 months of treatment, complete haematological response anti-CML (CHR, Fig. 1A) and >2log reduction of *BCR-ABL1/ABL1* ratio (down to 0.73%) were achieved albeit persistent lymphocytosis occurred (PB lymphocyte count >5.0 × 10^9^/L, Fig. 1A). At 10 months, lymphocytosis worsened despite the achievement of major molecular response (MMR; PB *BCR-ABL1/ABL1* ratio 0.02%; Fig. 1A) and further investigations revealed clonal expansion of karyotypically (Fig. 1B) and phenotypically (Fig. 1C) aberrant T-cells in PB and, later, in BM. Furthermore, monoclonal gamma-T-cell receptor gene rearrangement was detected in BM-derived DNA by PCR and low-level (10%) CD34-negative T-cell infiltration was found in BM whilst total body CT scan showed generalised lymphadenopathy. These findings together with histological examination of lymph node biopsy prompted the diagnosis of PTCL, not otherwise specified (NOS), and appropriate treatment commenced. First (CHOP-like chemotherapy) and second (IGEV poly-chemotherapy) line therapies failed. Instead, complete haematological and cytogenetic response of lymphoma was reached following the third line approach i.e. immunochemotherapy (Campath monoclonal antibody plus gemcitabine). During lymphoma treatment, imatinib was put on hold due to therapy/lymphoma-related myelosuppression without a negative effect on MMR of the CML. Considering the availability of a familial donor and refractory PTCL, the patient underwent a PB stem cell transplant (PBSCT). Donor granulocyte engraftment and complete remission of both haematological diseases lasted for six months after the procedure. Thereafter, progressive engraftment failure and the expansion of recipient haematopoiesis followed. The patient died at 11 months from PBSCT due to respiratory failure. Figure 1A summarises the clinical and treatment history of the patient.

## Results

**Figure 2:**
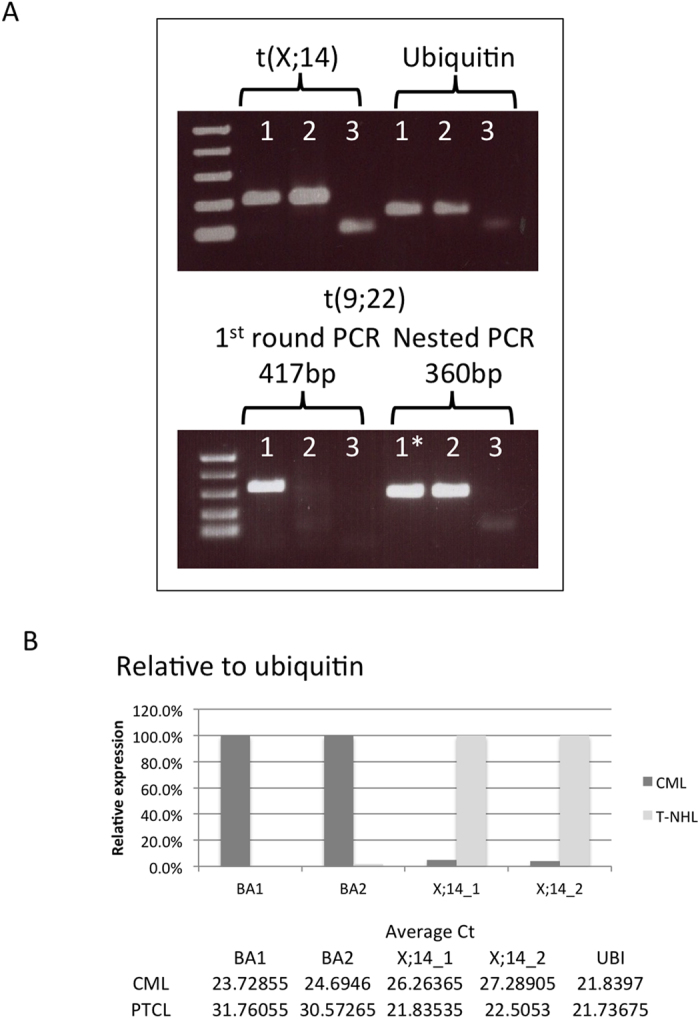
Molecular study of the presence of *BCR-ABL1* and t(X;14)-derived fusion transcripts in diagnostic samples corresponding to the two malignancies. (**A**) End-point qualitative PCR for the translocation product fusions using primers that map up and down stream of the breakpoints. For t(9;22) also nested PCR was performed. Sample legend: 1 – CML (diagnostic PB sample), 2 – T-NHL (diagnostic PB sample), 3 – H_2_O, * – 1^st^ round PCR product diluted 1:100 before being used as template in nested PCR. The bands visible in the no-template control lanes are due to primer dimer. (**B**) Quantitative PCR for the fusion gene transcripts. A graph of relative expression and tables of average C_t_s are shown. The relative quantity was calculated considering the amount of *BCR-ABL1*, normalised to ubiquitin, as 100% in the diagnostic CML sample and t(X;14) translocation product 100% in the diagnostic lymphoma sample.

**Figure 3:**
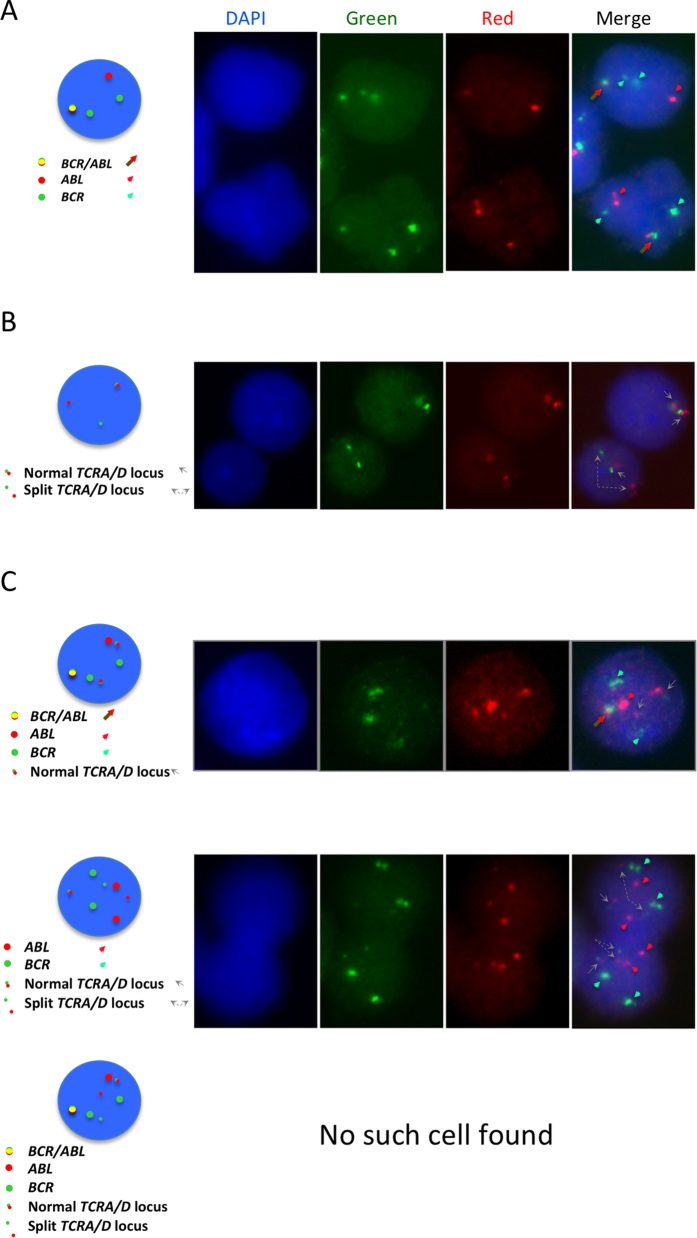
FISH characterisation of diagnostic CML and PTCL samples using the commercial probes that recognise the *BCR/ABL* fusion or the *TCRA/D* split. (**A**) Ideogram and a typical example of the pattern observed in pathological nuclei hybridised with the Vysis LSI BCR/ABL DC/DF probe showing one fusion, two green and one red signals corresponding to the loss of the reciprocal *ABL* locus. (**B**) Ideogram and a typical example of the pattern seen in lymphoma cells the bear the t(X;14) detected as split in the *TCRA/D* locus. A normal cell with intact 14q locus as testified by the vicinity of the red and green signals and an abnormal cell in which one of the two red/green pairs is separated due to the 14q break are shown. (**C**) Experiments performed combining the two probes. Big red signal corresponds to the *ABL* locus, the big green one to the *BCR* locus, whist the little red and green hybridisation spots correspond to the *TCRA/D* locus. Ideograms and examples of hybridisation are shown as well as an ideogram of an inexistent cell bearing both translocations.

The BCR-ABL1 protein found in the patient was of the p210 type and the breakpoints were ascertained by PCR with primers BCR-b1-A and ABL-a3-B (all primer sequences are given in Table 1). The finding of an amplicon of 417bp identified the transcript as b3-a2. Conventional cytogenetic analysis, performed at presentation of the second malignancy, has disclosed the presence of a t(X;14)(q28;q11) in the lymphoma cells. This translocation has been previously described to result in the breakage of the *BRCC3* gene and its fusion to the T-cell receptor alpha or delta (*TCRA/D*) locus on chromosome 14. We confirmed by PCR amplification and subsequent sequencing the involvement of the two loci. Next, in order to track the origins of the lymphoma, we designed primers (primers X;14_F1, X;14_R1) mapping up and down-stream of the fusion junction and applied them to cDNA retrotranscribed from mRNA sample extracted at the time of CML diagnosis. The end point-qualitative PCR experiments showed that the transcript deriving from the t(X;14) translocation fusion was present at the time of the first diagnosis (Fig. 2A, top panel). In parallel, we have looked for the *BCR-ABL1* fusion in mRNA deriving from PB sample taken at the time of PTLC diagnosis and found *BCR-ABL1* transcript in it (Fig. 2A, bottom panel) although at very low levels (primers BCR-b1-A and ABL-a3-B). The specificity of the faint band seen in the lane corresponding to the PTCL diagnostic sample was confirmed in a nested PCR protocol (primers BCR-b2-C and ABL-a3-D). Thus, we quantified the transcripts of the two translocations by qRT-PCR using two primer pairs for each fusion (qBCR_F1, qABL_R1, qBCR_F2, qABL_R2, X;14_F1, X;14_R1, X;14_F2, X;14_R2) and the SybrGreen methodology on both diagnostic samples and found that the expression level of the t(X;14) product in the diagnostic CML sample was about 4% of the expression of the *BCR-ABL1* fusion transcript in the same sample, whereas the expression of *BCR-ABL1* in the diagnostic PTCL sample was more than 10 times less (Fig. 2B). Next, we enumerated the reciprocal tumour burden in the two diagnostic samples by performing interphase FISH on archival methanol/acetic acid-fixed bone marrow samples taken at the times of CML and PTCL diagnoses. We used commercial probes designed to detect the presence of *BCR/ABL* fusion or *TCRA/D* locus breakage on the chromosome 14 caused by the t(X;14) formation. The experiments with the Vysis LSI BCR/ABL DC/DF probe (Abbott Molecular Inc., Des Plaines, IL, USA) disclosed the presence of an atypical hybridisation pattern consisting of one fusion signal, two green and one red signal (Fig. 3a). Such pattern has previously been reported and corresponds to a microdeletion of the reciprocal ABL locus. Cells with *BCR/ABL* fusion signal constituted 82% of the nuclei scored in the diagnostic CML sample. Not a single *BCR/ABL*+ cell was seen in the diagnostic PCTL sample when scoring a 1000 cells. FISH experiments with the use of TCRA/D Break-apart probe (Cytocell, Cambridge, UK) showed that the diagnostic CML sample contained about 4.6% (48/1028) of cells with the rearrangement in the *TCRA/D* locus (Fig. 3B), whilst at the time of PTCL diagnosis the percentage of such cells increased to 83%. We next applied the two probes together taking advantage of the fact that there is a substantial difference in size between the two, with probes for *BCR* and *ABL* being four times bigger than the *TCRA/D* Break-apart ones (600 kb vs ~150 kb). Cells with BCR/ABL fusion and no *TCRA/D* rearrangement as well as cells with *TCRA/D* split and no *BCR/ABL* fusion were identified in percentages equal to the ones seen for single-probe experiments. No cells with both abnormalities were encountered in the diagnostic CML sample (Fig. 3C).

## Discussion

Second malignancies diagnosed in patients previously treated for cancer can represent *de novo* unrelated oncogenic processes or be related/induced by the treatment received. Advances in diagnostics as well as in curative and supportive therapy have increased the number of long-term cancer survivors that may be prone to the development of secondary tumours arising from normal tissue exposure to the damaging effects of ionizing radiation or genotoxic stress of chemotherapy^8^,^9^,^10^,^11^. Recently, reports are emerging of additional secondary premalignant and malignant events, including *RAS*-mutant leukaemia, in melanoma patients on BRAF-inhibitors^12^. In particular, the patterns of incidence and latency of secondary lymphomas are distinct from that of myeloid malignancies and solid cancers. Indeed, the risk of secondary lymphomas increases after the first 5 years of completion of chemotherapy or radiotherapy and persists for more than three decades^13^. The lymphocytosis observed in our patient as soon as 3 months since the beginning of treatment with imatinib did not follow this pattern and prompted us to study the temporal relationship between the two malignancies. Interestingly, the occurrence of mono-/oligoclonal T-cell- or NK-cell- large granular lymphocyte lymphocytosis has been reported in a proportion of patients treated with dasatinib and to a lesser extent in those treated with imatinib^14^.

In order to answer the question of whether the two diseases manifested in the case presented here were genetically related or a coincidence, we took advantage of the presence of disease-specific translocations as clonal markers and looked for the presence of sequences derived from the respective fusions in the reciprocal diagnostic samples. The end point-qualitative PCR experiments showed that the transcript deriving from the t(X;14)(q28;q11) translocation fusion was already present at the time of the first diagnosis suggesting that a small clone of lymphoma cells existed 10 months before the finding of an overt lymphoma or that a small subclone of *BCR-ABL1* bearing cells existed in which t(X;14) was also present. However, FISH experiments confirmed that there were no cells in which the two translocations co-existed, i.e. PTCL cells are BCR/ABL negative. This is inconsistent with the theory of a subclone of CML evolving into PTCL as one would expect that such clonal evolution resulted in a cell that continued to bear t(9;22) and express BCR-ABL1. There is a slight discrepancy between the results of qPCR and FISH data when it comes to the evaluation of the CML burden at the time of PTCL diagnosis. The explanation for it includes several issues – the transcript levels/cell and the efficiency of PCR for the two primer pairs may be different in the two clones, the C_t_s were high and therefore outside of the range of accurate quantification. Nonetheless, there is no doubt as to the presence of residual *BCR-ABL1* transcript at the time of PTCL diagnosis, as diagnostic and nested protocols showed its presence.

The novelty of the case described here lies also in the finding of the t(X;14)(q28;q11) translocation in PTCL. This translocation, together with the more frequent inversion of the chromosome 14, has been previously described in T-cell prolymphocytic leukaemia^15^, admittedly, a disease from within the same spectrum of lymphoproliferations. PTCL NOS constitutes a rare and aggressive tumour whose molecular pathogenesis is still challenging^16^, albeit shedding light on the tumourigenesis of this disease entity is beyond the scope of this report.

Although secondary tumours have been described after imatinib therapy, the analyses of their frequency with respect to the age-adjusted general population yielded conflicting results^3^,^4^,^17^,^18^,^19^. Differences in cohort size, follow-up time and the interpretation of the term “secondary cancer” may have been responsible for this difference. Moreover, many of the above mentioned studies analysed historical data from cancer registries selecting patients treated with diverse modalities over variable periods of time. Clearly, working with historical data, especially if old, gives no opportunity of reviewing and confirming the diagnosis, anamnesis and so on. In the most recent article on the incidence of second malignancies following treatment with TKIs, Gunnarsson *et al.*^19^ reported an increased risk of a second cancer for the population of CML sufferers, indicating that such risk may more likely be related to the CML itself and not to the therapy with imatinib or other inhibitors. Without doubt, TKIs have dramatically changed the prognosis of patients with CML and are considered safe, well tolerated and non-mutagenic^2^. Our study suggests that in some cases the second malignancy is not secondary to TKIs treatment but instead was present at the time of diagnosis of CML. In such cases, the eradication of CML with the use of TKIs may create an opportunity for the lymphoma to develop overtly. We underline the importance of investigating the possibility that the second cancer in the context of a previous CML diagnosis was pre- or co-existent.

## Methods

We obtained informed consent from the patient prior to sample collection. The *ipsogen* BCR-ABL1 Mbcr IS-MMR Kit (Qiagen, Hilden, Germany) was used according to manufacturer’s instructions. Immunophenotypic/cytogenetic analyses of CML and PTCL were performed following standard diagnostic procedures and were carried out in accordance with the guidelines approved by Ethics Committee of the Istituto Europeo di Oncologia, Milan, Italy. Primers listed in Table 1 were used for molecular characterisation of breakpoints and specific transcript detection. Standard PCR reactions were performed with Taq polymerase obtained from New England Biolabs (Ipswich, MA, USA). qPCR was done utilising the SybrGreen (Applied Biosystems, Foster City, CA, USA) technology on the 7500 Fast Real-Time PCR System (Applied Biosystems) machine. Vysis LSI BCR/ABL Dual Color/Dual Fusion probe (Abbott Molecular Inc., Des Plaines, IL, USA) and TCRA/D Locus Break-apart probe (Cytocell, Cambridge, UK) were used according to manufactures’ instructions on methanol/acetic acid-fixed material previously used for conventional cytogenetic analysis. For infrequent cells (<10%) a total of 1000 cells were scored, whilst for frequent ones the scoring stopped at 200 nuclei. Slides hybridised with single probes were scored on the Olympus BX51 microscope (Olympus Corporation, Tokyo, Japan) equipped with the UPlanSApo 100X/1.40 oil, ∞/0.17/FN26.5 and a FITC/Texas Red double-band pass filter (Chroma Technology Corp®, Bellows Falls, VT, USA). Slides hybridised with the two probes together were analysed by capture on the Olympus BX61 fitted with the UPlanApo 100x/1.35 oil Iris, ∞/0.17, separate DAPI, FITC and wide-band red fluorescence (U-WMIY2) filters and CoolSNAP EZ CCD Camera (Photometrics, Tucson, AZ, USA). The capture was driven by the MetaMorph Microscopy Automation and Image Analysis Software (Molecular Devices, LLC, USA). Representative single colour images were saved as TIFF files and processed with the ImageJ tool devised by Wayne Rasband (wayne@codon.nih.gov, Research Services Branch, National Institute of Mental Health, Bethesda, MD, USA).

## Additional Information

**How to cite this article**: Gruszka, A. M. *et al.* Molecular investigation of coexistent chronic myeloid leukaemia and peripheral T-cell lymphoma - a case report. *Sci. Rep.* **5**, 14829; doi: 10.1038/srep14829 (2015).

## Figures and Tables

**Table 1:** Primers used for the study.

Primer name:	Sequence (5′–>3′)	Use
BCR-b1_A*	GAAGTGTTTCAGAAGCTTCTCC	End-point qualitative PCR
ABL-a3_B*	GTTTGGGCTTCACACCATTCC	
BCR-b2_C*	CAGATGCTGACCAACTCGTGT	Nested end-point qualitative PCR
ABL-a3-D*	TTCCCCATTGTGATTATAGCCTA	
qBCR_F1	GATGCTGACCAACTCGTGTG	End-point qualitative PCR, qRT-PCR
qABL_R1	CGCTGAAGGGCTTCTTCCTTA	
qBCR_F2	TCCAGACTGTCCACAGCATTC	qRT-PCR
qABL_R2	CCTTGGAGTTCCAACGAGCG	
X;14_F1	TCGGTGAATAGGCAGACAGAC	qRT-PCR
X;14_R1	CTCGAGTCTGACGCTTTCCT	
X;14_F2	GCAGACAGACTTGTCACTGGA	qRT-PCR
X;14_R2	CGTTTGTCTCAACCACGCTC	

^*^Primer sequence published in^20^.
